# Seasonal Occurrence and UV-Based Oxidative Removal of Odorants in Surface Water: Insights into Structure-Dependent Reactivity

**DOI:** 10.3390/toxics14080698

**Published:** 2026-08-06

**Authors:** Junkai Feng, Congcong Li, Hao Jiao, Xiaodong Xin, Ruibao Jia, Weiqiang Zhu

**Affiliations:** 1 School of Water Conservancy and Environment, University of Jinan, Jinan 250022, China; 2 Jinan Municipal Engineering Design & Research Institute (Group) Co., Ltd., Jinan 250102, China; 3 Shandong Provincial Water Supply and Drainage Monitoring Centre, Jinan 250101, China

**Keywords:** odorants, Pearson correlation, seasonal occurrence, structure-activity relationship, UV-AOPs

## Abstract

Odorants present a persistent challenge in drinking water treatment due to their low odor threshold concentrations (OTCs) and their resistance to conventional processes. This study investigated the seasonal occurrence of eight odorants across 20 surface water sources in Shandong, China. It evaluated their degradation using UV photolysis and four UV-based advanced oxidation processes (UV-AOPs): UV/H_2_O_2_, UV/PMS, UV/Cl, and UV/TiO_2_. Total odorant concentrations ranged from 8 to 496 ng L^−1^ in the wet season and from 5 to 184 ng L^−1^ in the dry season, with 2-MIB as the dominant compound (detection frequency: 95%; contributions: 58.48% and 48.88%, respectively). Pearson correlation analysis revealed a seasonal shift in environmental drivers: total phosphorus (TP) and dissolved oxygen (DO) in the wet season versus NH_3_-N and COD_Mn_ in the dry season. Across all five processes, degradation efficiency followed a consistent, structure-dependent hierarchy, ranking as CTA >> 2,4,6-TCA ≈ IPMP > IPO > 2-MIB ≈ GSM, with CTA achieving 74.66–95.03% removal while 2-MIB and GSM remained largely refractory (≤29.70%). Process-specific selectivity was also observed: UV/H_2_O_2_ was most effective for IPO, IPMP, and GSM; UV/PMS excelled for CTA and 2,4,6-TCA; and UV/Cl showed a slight advantage for 2-MIB. These findings provide mechanistic insights into structure-dependent odorant oxidation and suggest that UV-AOP performance may vary according to target-compound characteristics. Further validation in real water matrices is required before practical application.

## 1. Introduction

Reservoirs, which constitute a major component of China’s surface freshwater resources, are increasingly affected by cyanobacterial blooms and eutrophication [1,2]. Among the resulting water quality issues, taste and odor (T&O) problems are particularly pervasive. A nationwide survey indicated that odors were present in 80% of raw water and 45% of finished drinking water samples [3], and well-documented incidents in Wuxi [4] and Hangzhou [3] underscore the potential for public health emergencies and widespread consumer distrust.

Most T&O compounds originate as algal or actinomycetal metabolites. Among these, geosmin (GSM) and 2-methylisoborneol (2-MIB), which are known for their earthy–musty odor, are the most frequently detected and studied [5]. Other compounds, such as 2-isopropyl-3-methoxypyrazine (IPMP) and 2-isobutyl-3-methoxypyrazine (IBMP), which evoke rotten vegetable odors, have been detected in freshwater lakes at concentrations of 10–65 ng L^−1^ [6,7]. Additionally, 2,4,6-trichloroanisole (2,4,6-TCA), a chlorination byproduct formed via microbial O-methylation of 2,4,6-trichlorophenol in distribution systems, poses substantial risks owing to its exceptionally low odor threshold [8]. Beyond aesthetic concerns, many of these compounds may cause respiratory irritation, endocrine disruption, and digestive dysfunction upon chronic exposure [9,10,11]. Effective removal of these trace contaminants is therefore essential for ensuring both drinking water acceptability and consumer safety.

Conventional treatment processes (coagulation, sedimentation, and filtration) remove these odorants poorly, especially at sub-µg L^−1^ levels, due to their low concentrations and hydrophilic properties [12]. The co-occurrence of natural organic matter (NOM) further exacerbates this inefficiency by competing for adsorbent surfaces and consuming oxidants [13]. To address these limitations, UV-based advanced oxidation processes (UV-AOPs), such as UV/H_2_O_2_, UV/PMS, UV/Cl, and UV/TiO_2_, have attracted increasing attention for T&O control due to their fast kinetics, mild operating conditions, and strong radical generation [14,15].

Despite these advances, several critical knowledge gaps remain. Most previous studies have focused on one or two representative odorants (typically 2-MIB or GSM) under simplified oxidation schemes, leaving the multicomponent mixtures characteristic of actual source waters largely unexplored [14,16]. Consequently, field data documenting the seasonal co-occurrence and concentration profiles of diverse odorant classes, ranging from cyclic alcohols to pyrazines and anisoles, across regional water supplies are scarce. Furthermore, a systematic head-to-head comparison of different UV-AOPs for structurally dissimilar odorants under identical experimental conditions has not been performed [16,17], hindering the establishment of robust structure-reactivity correlations. Perhaps more critically, how molecular architecture dictates oxidative susceptibility remains poorly understood [14,18], limiting the development of predictive frameworks for process selection based on source water composition.

To address these gaps, this study presents a comprehensive investigation integrating field monitoring with bench-scale process comparison. The specific objectives were to: (1) investigate the seasonal occurrence and distribution patterns of eight typical odorants (2-MIB, GSM, IPMP, IBMP, 2,4,6-TCA, 2,3,6-TCA, 2,3,4-TCA, β-cyclocitral (CTA), β-ionone (Ionone), and isophorone (IPO)) across 20 surface water sources in Shandong Province, China, during both wet and dry seasons; (2) elucidate the correlations between odorant concentrations and key water quality parameters using Pearson correlation analysis to infer potential environmental drivers; (3) evaluate the removal efficiencies and apparent pseudo-first-order degradation kinetics of six representative odorants (2-MIB, GSM, IPO, IPMP, CTA, and 2,4,6-TCA) under five predefined UV-based treatment conditions, including UV photolysis, UV/H_2_O_2_, UV/PMS, UV/Cl, and UV/TiO_2_, and identify compound- and process-dependent degradation patterns without inferring a molar-normalized ranking of intrinsic process efficiency; and (4) discuss the influence of molecular structure on oxidation behavior and provide process selection guidelines for drinking water utilities. These findings are expected to inform adaptive, target-oriented odor control strategies.

## 2. Materials and Methods

### 2.1. Chemicals and Reagents

Analytical-grade standards of GSM, 2-MIB, IPMP, IBMP, 2,4,6-TCA, 2,3,4-TCA, 2,3,6-TCA, CTA, Ionone, and IPO were used. H_2_O_2_ (30%, *w*/*w*), potassium peroxymonosulfate (PMS, 2KHSO_5_·KHSO_4_·K_2_SO_4_, 42–46% KHSO_5_), TiO_2_ (99.8%, particle size 25 nm), NaClO, and HPLC-grade methanol and dichloromethane were obtained from commercial suppliers. All solutions were prepared with ultrapure water (Milli-Q, 18.2 MΩ·cm). A mixed stock solution of the target odorants in methanol was stored at 4 °C in the dark, and working solutions were freshly diluted with ultrapure water before use.

### 2.2. Sample Collection

Surface water samples were collected from 20 major reservoirs and water sources across Shandong Province, China, during the wet (July–August) and dry (November–December) seasons. The geographical distribution of the sampling sites is illustrated in Figure S1 of the Supplementary Material. At each site, water was collected from 0.5 m below the surface using brown glass bottles filled without headspace to minimize volatilization. Samples were immediately labeled, transported on ice to the laboratory, and stored at 4 °C in the dark. All physicochemical and odorant analyses were completed within one week. Field blanks and parallel samples were included for quality assurance and control (QA/QC).

### 2.3. Oxidation Degradation Experiments

#### 2.3.1. Experimental Setup

Degradation experiments were conducted in a bench-scale UV photochemical reactor (BILON-GHX-ID, Figure S2 of the Supplementary Material) equipped with a magnetic stirrer and eight cylindrical quartz tubes (100 mL each). A 200 W high-pressure mercury lamp (365 nm, 0.18 mW cm^−2^) served as the UV source. The 365 nm wavelength was chosen to mimic near-UV conditions relevant to practical water treatment, and the lamp output was held constant across all runs to ensure inter-process comparability.

Based on field occurrence data and structural diversity, six representative odorants (2-MIB, GSM, IPO, IPMP, CTA, and 2,4,6-TCA) were selected as targets. Mixed working solutions were prepared in ultrapure water at a concentration of 1 μg L^−1^ for each compound, a level representative of elevated field concentrations and suitable for reliable kinetic quantification. Reactions were conducted at room temperature (25 ± 1 °C) without pH adjustment (initial pH~6.8), and solutions were stirred for 10 min before UV exposure to ensure homogeneity.

#### 2.3.2. UV Photolysis and UV-AOPs Procedures

Five UV-based treatments were evaluated, including UV photolysis, UV/H_2_O_2_, UV/PMS, UV/Cl, and UV/TiO_2_. The initial dosages were 8 mg L^−1^ H_2_O_2_, 5 mg L^−1^ PMS, 5 mg L^−1^ free chlorine, and 200 mg L^−1^ TiO_2_. These dosages were selected from preliminary trials and previously reported laboratory conditions [16,19] to provide measurable degradation within the experimental period. The applied concentrations were not normalized on an equimolar, oxidant-equivalent, or radical-exposure basis. Therefore, the experiments were designed as a comparative screening of odorant degradation behavior under the specified operating conditions rather than a quantitative comparison of intrinsic process efficiency or oxidant utilization. In addition, TiO_2_ was applied as a heterogeneous photocatalyst loading and was therefore not directly comparable with the dissolved oxidants on a molar basis.

In each run, 100 mL of the mixed odorant solution was transferred to a quartz tube, and the corresponding oxidant or catalyst was added just before UV irradiation. The solution was stirred continuously throughout. At 0, 20, 40, 60, 80, and 100 min, 5 mL aliquots were withdrawn and immediately quenched with 50 μL of methanol to stop radical reactions. For UV/TiO_2_, samples were filtered through 0.22 μm PVDF syringe filters before analysis to remove catalyst particles. All experiments were conducted in duplicate, and results are reported as averages with standard deviations.

### 2.4. Analysis Methods

#### 2.4.1. Solid-Phase Extraction (SPE) of Odorants

Prior to GC–MS analysis, 100 mL water samples were preconcentrated using an automated SPE system (Auto SPE 06PLUS (Reeko, Xiamen, China)) with HLB cartridges (6 cc, 200 mg, Waters, Milford, MA, USA). The conditioning of HLB cartridges was performed sequentially with 5 mL methanol and 10 mL ultrapure water at 10 mL min^−1^. After loading 100 mL of sample at the same flow rate, the cartridges were rinsed with 5 mL of ultrapure water and dried under nitrogen for 25 min. The analytes were then eluted with 2.5 mL of dichloromethane at 3 mL min^−1^, and the eluates were concentrated to near dryness under a gentle nitrogen stream before being reconstituted in 1 mL of methanol for GC–MS analysis. The detailed SPE protocol is given in Table S1 of the Supplementary Material.

#### 2.4.2. Odorant Quantification by GC–MS

Target odorants in both field samples and degradation experiments were quantified using a GC–MS system (TRACE-DSQ, ThermoFisher Scientific (Waltham, MA, USA)) equipped with a DB-5 MS capillary column (30 m × 0.25 mm i.d. × 0.25 μm film thickness) (Agilent Technologies, Santa Clara, CA, USA). The injection volume was 1.0 μL in splitless mode. The mass spectrometer was operated in electron impact (EI) mode at 70 eV with the ion source at 230 °C. Quantification used selected ion monitoring (SIM) based on characteristic m/z ratios for each compound (retention times and ions listed in Table S2). External calibration curves (R^2^ > 0.995) were constructed over 0.1–500 μg L^−1^, with limits of detection (LODs) ranging from 0.1 to 0.5 ng L^−1^ for the target compounds.

#### 2.4.3. Water Quality Parameters

Routine water quality indicators, including pH, dissolved oxygen (DO), permanganate index (COD_Mn_), NH_3_-N, total nitrogen (TN), and total phosphorus (TP), were determined according to standard methods [20]. In oxidation experiments, H_2_O_2_ was quantified with a portable detector (GDYS-102SC), and residual free chlorine was determined by the DPD colorimetric method.

### 2.5. Kinetic Analysis

Degradation kinetics were evaluated by fitting the experimental data to a pseudo-first-order model, which is widely used for trace organic contaminant oxidation in UV-based systems [21,22]:

(1) $$-\frac{d\left[C\right]}{dt}=k_{obs}\cdot \left[C\right]$$

(2) $$\ln\left(\frac{c_{0}}{c_{t}}\right)=k_{obs}\cdot t$$

where C_0_ and C_t_ (μg L^−1^) are the odorant concentrations at time 0 and time t (min), respectively, and k_obs_ (min^−1^) is the apparent pseudo-first-order rate constant. Rate constants were obtained by linear regression of ln(C_0_/C_t_) versus t, and only data with R^2^ ≥ 0.90 were included.

## 3. Results and Discussion

### 3.1. Occurrence and Seasonal Variation of Odorants in Source Waters

#### 3.1.1. Concentration Levels and Detection Frequencies

**Figure 1:**
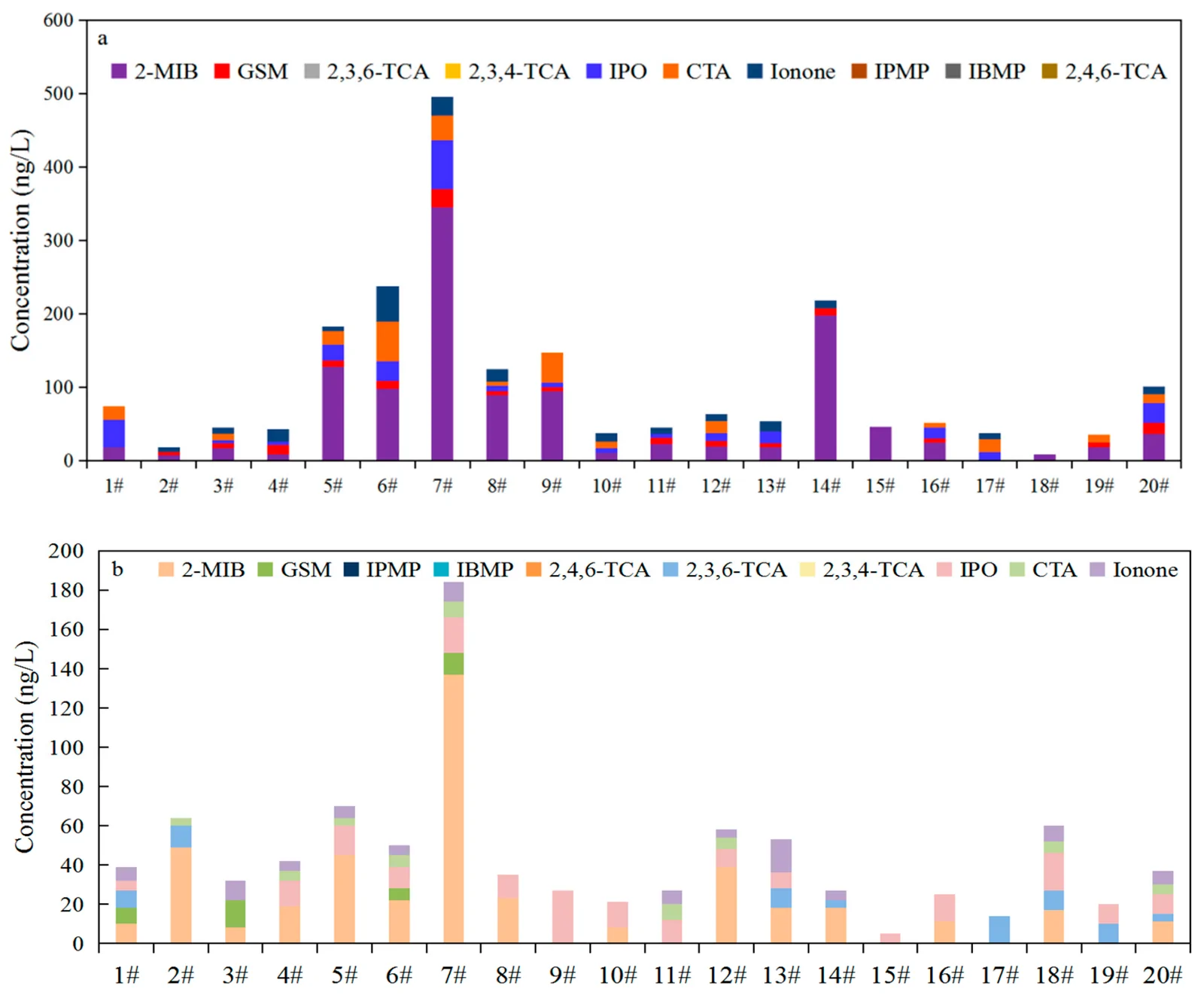
Concentrations and distributions of odorants in each reservoir during (**a**) the wet season, and (**b**) the dry season. The symbol ‘#’ denotes the sampling point numbers, and the specific locations of each sampling point are shown in Figure S1 of the Supplementary Material.

The concentrations and detection frequencies of eight target odorants across 20 sampling sites during the wet and dry seasons are summarized in Figure 1. In the wet season, total odorant concentrations ranged from 8 to 496 ng L^−1^, with 2-MIB being the most abundant compound, accounting for 58.50% of the total burden and exhibiting a detection frequency of 95%. GSM, IPO, CTA, and Ionone also showed detection rates above 65%, with average contributions of 6.59%, 12.98%, 12.02%, and 9.95%, respectively. The prevalence of 2-MIB and GSM is consistent with previous reports on cyanobacteria-dominated waters in China [23,24,25], reflecting the widespread occurrence of these microbial metabolites in eutrophic freshwater systems.

In the dry season, total odorant concentrations decreased to 5–184 ng L^−1^, and only six of the eight target compounds were detected (IPMP and IBMP were absent). 2-MIB remained the dominant contributor (48.88%), followed by IPO (22.58%) and Ionone (10.22%). The detection frequencies of most odorants were lower in the dry season, with GSM and TCA dropping to 20% and 40%, respectively. This seasonal decline is consistent with reduced cyanobacterial activity under lower temperatures and diminished hydrological inputs during the dry period [26].

#### 3.1.2. Seasonal Odor Risk Assessment

Based on the occurrence data, we further assessed the potential sensory risks posed by these odorants. The odor threshold concentrations (OTCs) of the target compounds range from sub-ng L^−1^ to tens of ng L^−1^ (Table 1) [27,28,29]. In the wet season, 2-MIB concentrations (7–345 ng L^−1^) exceeded its OTC (5–10 ng L^−1^) at the majority of sites, indicating a substantial risk of earthy-musty odor episodes. GSM, IPO, CTA, and Ionone were also detected at levels surpassing their respective OTCs at several locations. Even in the dry season, when concentrations were generally lower, 2-MIB and IPO still exceeded their OTCs at multiple sites. These findings suggest that odor risk is not confined to bloom seasons but persists year-round, warranting seasonal-adaptive management strategies.

#### 3.1.3. Correlations with Conventional Water Quality Parameters

**Figure 2:**
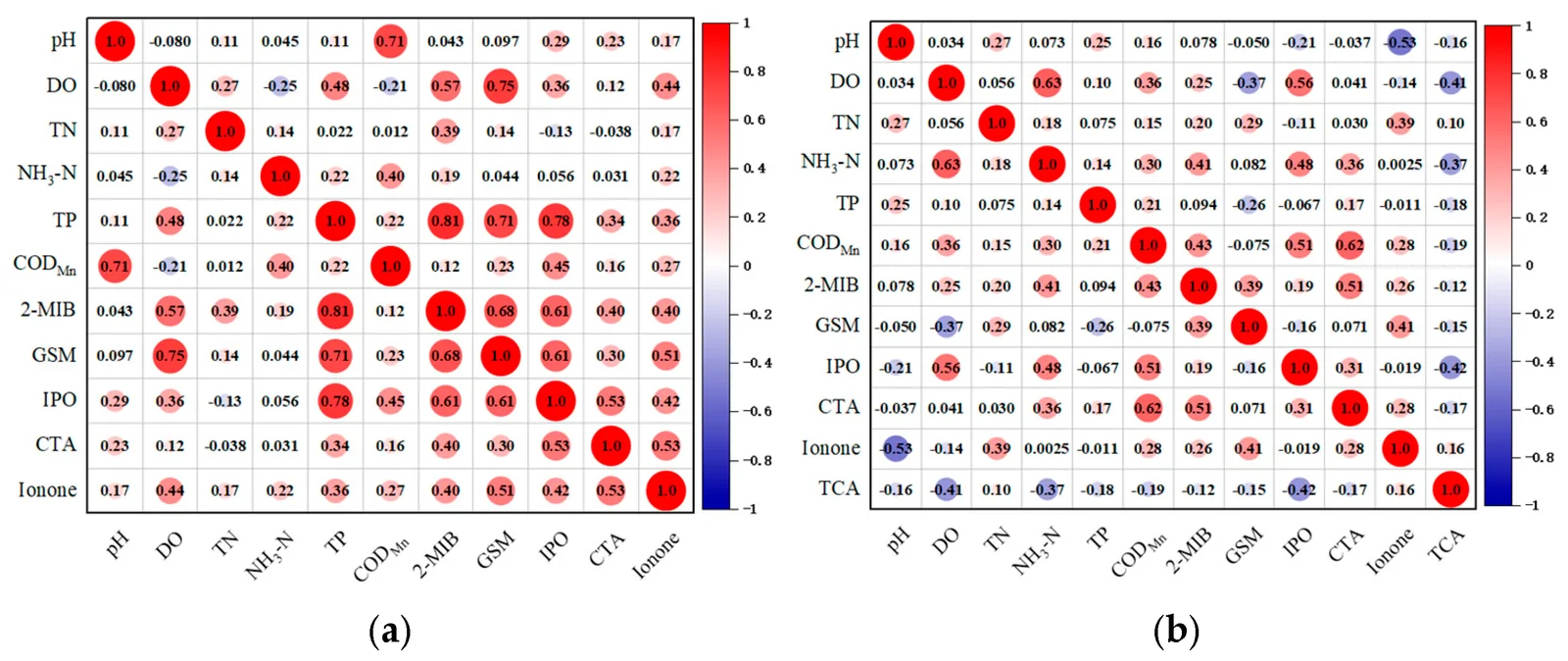
Correlation matrices of odorants and conventional water quality parameters. (**a**) Wet season; (**b**) Dry season. Color intensity and numbers indicate Pearson correlation coefficients (r). Positive and negative correlations are shown in red and blue, respectively.

Pearson correlation analysis (Figure 2) revealed distinct seasonal patterns in the associations between odorants and water quality indicators. In the wet season, 2-MIB, GSM, IPO, CTA, and Ionone were positively correlated with TP (r = 0.34–0.81) and DO (r = 0.12–0.75). The positive correlations of odorants with TP and DO in the wet season suggest that algal-mediated production is the dominant pathway during this period. This is consistent with phosphorus being a key limiting nutrient for cyanobacterial growth and DO serving as a proxy for photosynthetic activity [30,31].

In contrast, the dry season correlations shifted toward NH_3_-N and COD_Mn_. IPO, 2-MIB, and CTA were positively correlated with NH_3_-N (r = 0.36–0.48) and COD_Mn_ (r = 0.43–0.62). NH_3_-N is indicative of nitrogenous nutrient loading, while COD_Mn_ reflects overall organic pollution levels. Both parameters can influence the growth of odor-producing microorganisms and the accumulation of organic precursors [32]. Notably, GSM and TCA showed negative correlations with DO (r = −0.37 and −0.41, respectively) in the dry season, which may imply a shift in source dominance under reduced hydrological mixing and lower temperatures. Specifically, the dominant source shifts from in-lake production to sediment release or anaerobic degradation.

Overall, these results indicate a seasonal shift in the environmental drivers of odorant occurrence: from algal metabolism-related factors (TP, DO) in the wet season to nutrient accumulation and organic matter-related factors (NH_3_-N, COD_Mn_) in the dry season [30,32]. However, correlation analysis alone cannot establish causality, and future studies incorporating multivariate statistics and algal biomass data are needed to better resolve these interactions. The seasonal shift in correlation patterns also carries important implications for monitoring strategies. During wet seasons, indicators of algal productivity (TP, DO) may serve as effective early-warning proxies for odorant elevation [30,31], whereas in dry seasons, routine organic matter and nitrogen parameters (COD_Mn_, NH_3_-N) could better reflect odor risk. This dual-indicator framework offers a pragmatic approach for water utilities to adjust monitoring frequencies and trigger thresholds according to seasonal prevailing conditions.

### 3.2. UV-Based Advanced Oxidation Processes for Odorant Removal

#### 3.2.1. Overall Removal Performance

The removal efficiencies of the six odorants after 100 min under the five UV-based processes are summarized in (Table 2). To facilitate comparison of structure-dependent degradation behavior while minimizing matrix interference, the oxidation experiments were conducted under controlled ultrapure-water conditions. The results therefore represent apparent treatment responses under the specified operating conditions rather than intrinsic reactivity constants.

CTA was the most readily degraded (89.53–95.03%, except UV/TiO_2_ at 74.66%), reflecting the high reactivity of its conjugated enone structure toward both photolysis and radical attack. 2,4,6-TCA and IPMP showed moderate removals (43.92–73.17%), while IPO, 2-MIB, and GSM were poorly removed (< 35%) due to the absence of extended conjugation or favorable electrophilic sites. This ranking (CTA >> 2,4,6-TCA ≈ IPMP > IPO > 2-MIB ≈ GSM) remained consistent across all five processes, suggesting that molecular structure governs oxidative susceptibility more strongly than the choice of AOP [18,33,34]. The rate constants summarized in Table S3 of the Supplementary Material provide quantitative support for this hierarchy, which forms the basis for the mechanistic discussions that follow.

#### 3.2.2. UV Photolysis

**Figure 3:**
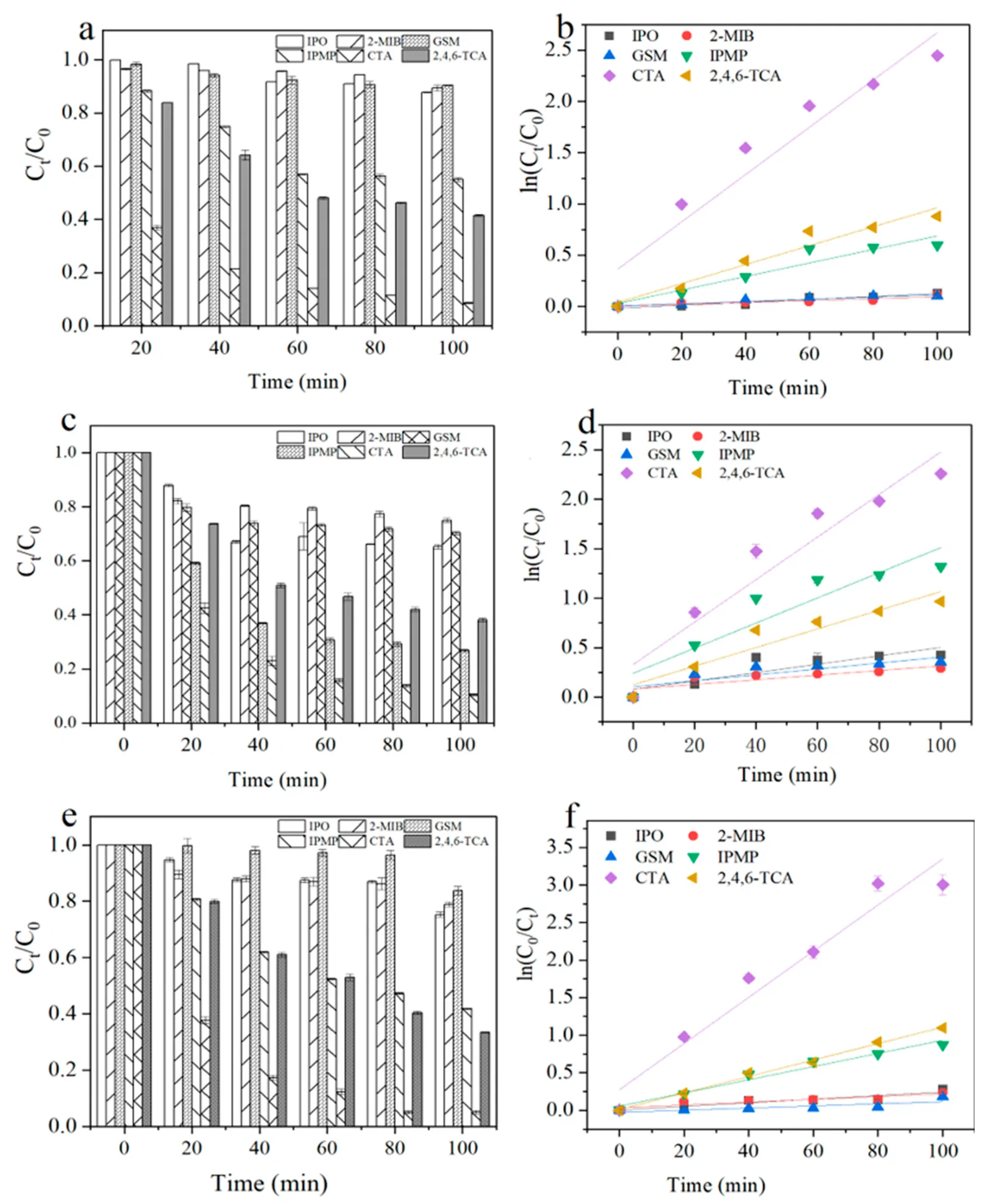
Removal efficiencies (**a**,**c**,**e**) and corresponding pseudo-first-order rate constants (**b**,**d**,**f**) for six odorants treated with UV photolysis (**a**,**b**), UV/H_2_O_2_ (**c**,**d**), and UV/PMS (**e**,**f**). Experimental conditions: [H_2_O_2_] = 8 mg L^−1^, [PMS] = 5 mg L^−1^, reaction time = 100 min.

UV irradiation alone removed CTA most efficiently (91.34%), followed by 2,4,6-TCA (58.53%) and IPMP (45.06%), while 2-MIB (10.66%), GSM (9.55%), and IPO (12.28%) showed limited degradation (Figure 3a,b). These results reflect the importance of molecular chromophores under our experimental conditions (365 nm). CTA, with its conjugated enone system (C=C–C=O), exhibits an n → π* transition in the near-UV region, while 2,4,6-TCA benefits from the aromatic ring’s π → π* absorption, and both of these transitions overlap with the 365 nm emission of the UV lamp [18,35].

In contrast, 2-MIB and GSM, being saturated cyclic alcohols, lack such low-energy electronic transitions, rendering them transparent at 365 nm and resistant to direct photolysis [18,36]. The correlation between chromophores and degradation efficiency highlights that direct photolysis is primarily effective for odorants with low-energy electronic transitions [18]. In this correlation, CTA and 2,4,6-TCA showed the highest removals, whereas 2-MIB and GSM exhibited the lowest.

#### 3.2.3. UV/H_2_O_2_ and UV/PMS

The performance of UV/H_2_O_2_ and UV/PMS is shown in Figure 3. The addition of H_2_O_2_ or PMS significantly enhanced the degradation of most odorants compared to UV photolysis alone, which verifies that photogenerated radicals substantially facilitate the overall elimination of these contaminants [37,38]. However, the extent of enhancement was highly structure-dependent, and a direct comparison reveals distinct selectivity patterns.

For CTA and 2,4,6-TCA, UV/PMS outperformed UV/H_2_O_2_ (95.02% vs. 89.53% and 66.62% vs. 61.88%), achieving the highest removals among all five processes. This superiority can be rationalized by the complementary reactivity of sulfate radicals (SO_4_∙^−^). In contrast to the indiscriminate hydrogen abstraction favored by ∙OH, SO_4_∙^−^ acts as a moderately electrophilic oxidant that exhibits preferential addition to electron-rich moieties [38]. For CTA, the conjugated enone system (C=C–C=O) provides a favorable site for SO_4_∙^−^ addition. For 2,4,6-TCA, although chlorine substituents exert an electron-withdrawing effect, the chlorinated aromatic ring retains sufficient π-electron density to undergo electrophilic attack [18,34]. In both cases, the initial radical addition likely triggers rapid ring-opening or side-chain cleavage, accounting for the near-complete removal of CTA under UV/PMS [33].

In contrast, UV/H_2_O_2_ demonstrated superior performance for IPO and IPMP (34.73% and 73.17% vs. 24.76% and 58.24% for UV/PMS). This reversal in selectivity suggests that the less selective but more reactive ∙OH is more effective at abstracting hydrogen atoms from the methoxy or isopropyl substituents on the pyrazine ring of IPMP, or from the isopropyl side chain adjacent to the carbonyl group in IPO [38]. The higher reactivity of ∙OH toward aliphatic C–H bonds, combined with its smaller size and lower steric demand, likely facilitates attack at these peripheral sites that are less accessible to the bulkier SO_4_∙^−^. Collectively, these results reveal a complementary selectivity pattern. UV/PMS favors electron-rich conjugated structures, whereas UV/H_2_O_2_ demonstrates greater effectiveness for compounds with aliphatic side chains that are susceptible to hydrogen abstraction [39].

For the saturated bicyclic tertiary alcohols 2-MIB and GSM, both radical systems performed poorly, with removals ranging from 16.25% to 29.70%. This recalcitrance reflects the absence of favorable reaction sites: their degradation relies almost exclusively on hydrogen abstraction from tertiary C–H bonds [36]. The marginal improvement with UV/H_2_O_2_ over UV/PMS (29.70% vs. 16.25% for GSM and 25.04% vs. 21.05% for 2-MIB) suggests that the smaller ∙OH radical partially overcomes the steric barrier of the compact bicyclic ring system. Rate constants (Table S3 of the Supplementary Material) support this: 2-MIB and GSM showed k_obs_ values of 4.23 × 10^−3^ and 2.33 × 10^−3^ min^−1^ under UV/H_2_O_2_, marginally higher than under UV/PMS (2.40 × 10^−3^ and 1.87 × 10^−3^ min^−1^), in sharp contrast to CTA (2.15 × 10^−2^ to 3.07 × 10^−2^ min^−1^) [33].

#### 3.2.4. UV/Cl

**Figure 4:**
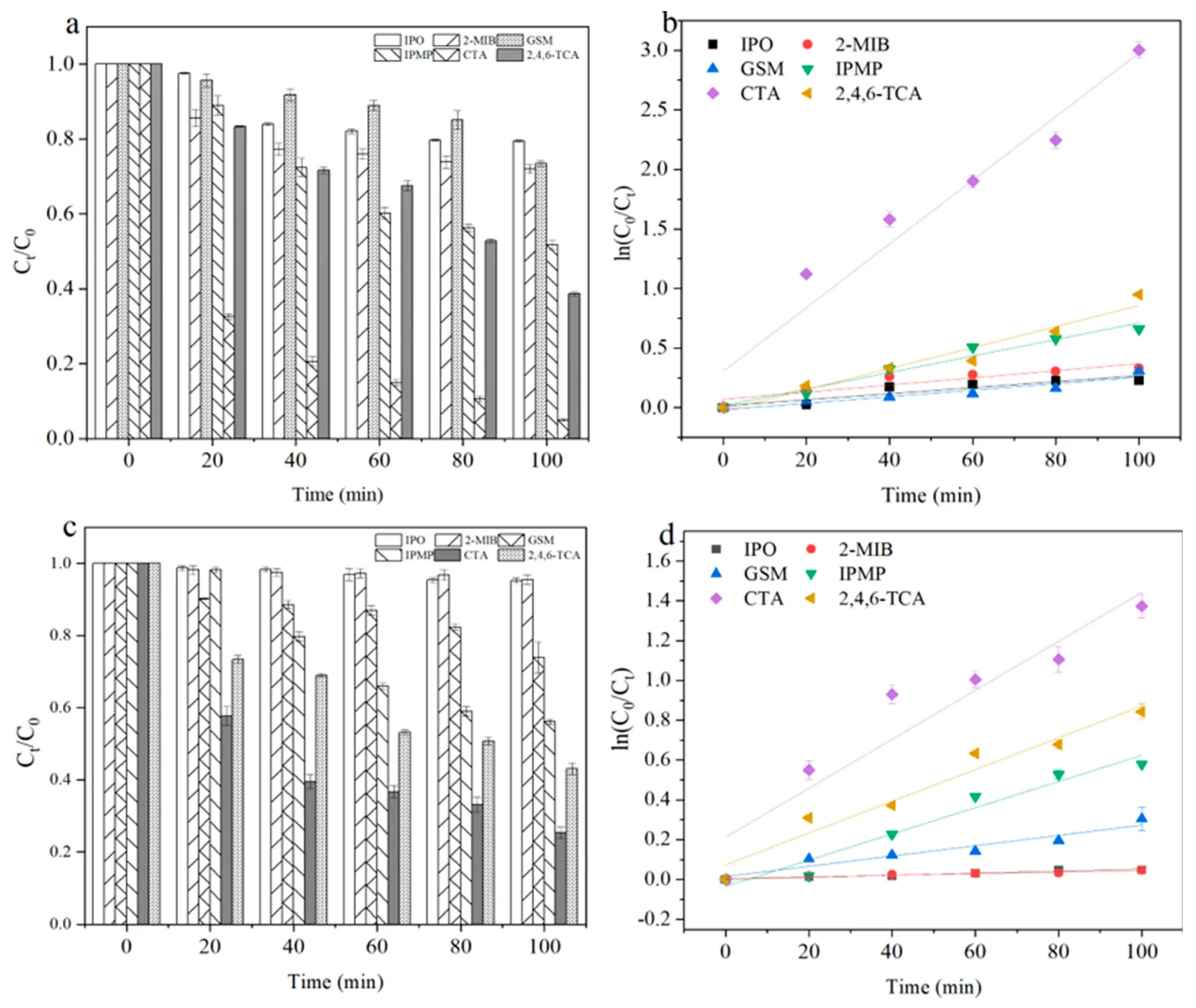
Removal efficiencies (**a**,**c**) and corresponding pseudo-first-order rate constants (**b**,**d**) for six odorants treated with UV/Cl (**a**,**b**) and UV/TiO_2_ (**c**,**d**). Experimental conditions: [free chlorine] = 5 mg L^−1^ (as NaClO), TiO_2_ dosage = 200 mg L^−1^ (20 mg in 100 mL), reaction time = 100 min.

UV/Cl also showed excellent removal of CTA (95.03%), outperforming all other processes for this compound, while moderately removing 2,4,6-TCA (61.31%) and IPMP (48.31%) (Figure 4a,b). The degradation in UV/Cl is driven by reactive chlorine species (RCS, e.g., Cl∙, Cl_2_∙^−^) and ∙OH generated from the photolysis of free chlorine [40,41]. The marginal advantage of UV/Cl for 2-MIB over the other AOPs likely arises from the dual radical chemistry of this system: while ∙OH provides broad-spectrum oxidation, the coexisting Cl∙ introduces additional selectivity toward the sterically accessible tertiary C–H center in 2-MIB [41].

Notably, UV/Cl achieved the highest 2-MIB removal (28.02%) among all processes, contrasting with its marginal performance for GSM (26.49%). It is hypothesized that Cl∙, being larger and more polarizable than ∙OH, exhibits higher selectivity for the tertiary C–H bond adjacent to the hydroxyl group in 2-MIB [35], while the greater steric congestion around GSM’s tertiary center hinders the bulkier Cl∙ approach. This compound-specific selectivity, however, does not overcome the intrinsic recalcitrance of saturated bicyclic compounds, as absolute removals remained below 30%. Additionally, UV/Cl poses a risk of disinfection by-product formation in waters containing bromide [42].

#### 3.2.5. UV/TiO_2_

Photocatalytic oxidation by UV/TiO_2_ exhibited relatively low removal efficiencies for most of the target odorants under the applied experimental conditions. After 100 min of irradiation, the removal efficiencies of 2-MIB and IPO were only 4.54% and 4.73%, respectively, both of which were lower than those obtained by UV photolysis alone. CTA removal reached 74.66%, but remained lower than that observed in the other UV-based systems, whereas GSM and 2,4,6-TCA showed removals of 26.08% and 56.97%, respectively (Figure 4c,d).

The relatively poor performance of UV/TiO_2_ was more plausibly associated with the optical and mass-transfer characteristics of the heterogeneous photocatalytic system than with competitive adsorption among the target odorants. At the applied TiO_2_ loading of 200 mg L^−1^, particle scattering and suspension turbidity may have attenuated photon penetration through the reaction solution, thereby reducing the effective irradiation of catalyst particles and limiting electron–hole pair generation within the reactor. Similar light attenuation and catalyst-loading effects have been widely reported in heterogeneous TiO_2_ photocatalytic systems, where excessive catalyst concentrations may decrease photon utilization efficiency due to increased light scattering and reduced effective irradiation of active sites [43,44].

In addition, photocatalytic degradation requires the transport of dissolved odorants from the bulk solution to reactive sites at or near the TiO_2_ surface. For compounds occurring at trace concentrations, this heterogeneous transport step may restrict their access to short-lived surface-associated reactive species, which is a recognized limitation in heterogeneous photocatalytic reactions [45]. The limited removals of 2-MIB and IPO may therefore reflect a combination of weak interfacial affinity, restricted mass transfer, and insufficient effective photon flux rather than competition among the coexisting odorants for surface-active sites. Because adsorption measurements, catalyst agglomeration, and photon-flux distributions were not directly determined in this study, these mechanisms should be regarded as plausible explanations rather than experimentally verified pathways.

Accordingly, the results indicate that UV/TiO_2_ was ineffective for most of the investigated odorants under the specific catalyst loading and reactor configuration employed here. This observation should not be interpreted as demonstrating the general unsuitability of TiO_2_ photocatalysis for odorant removal, because its performance may be improved through catalyst-dose optimization, enhanced suspension or immobilization strategies, and reactor designs that increase photon penetration and interfacial mass transfer.

#### 3.2.6. Mechanistic Insights into Structure-Activity Relationships

The reactivity hierarchy established above provides the basis for elucidating the molecular origins of differential treatability. Three structural attributes are key: chromophoric properties, electron density distribution, and steric accessibility.

Compounds bearing conjugated π-systems, such as CTA and 2,4,6-TCA, are indeed the most readily degraded among all tested odorants, with removal efficiencies of 74.66–95.02% and 56.97–66.62%, respectively, across all five UV-based processes. This consistently high reactivity can be attributed to the extended π-conjugation that reduces the HOMO-LUMO gap, facilitating electron transfer and rendering these compounds more susceptible to electrophilic attack by radicals [35]. Specifically, the conjugated carbonyl group in CTA and the chlorinated aromatic ring in 2,4,6-TCA serve as preferential sites for radical addition, leading to rapid ring-opening or side-chain cleavage as the initial degradation steps.

At the bottom of the reactivity ladder lie the saturated bicyclic tertiary alcohols (2-MIB and GSM). Lacking both chromophores and π-electrons, these compounds are resistant to direct photolysis and electrophilic radical addition. Their degradation is largely confined to hydrogen abstraction from tertiary C–H bonds, a route substantially impeded by the steric constraints of the fused bicyclic ring system and the adjacent methyl substituents [36]. This universal recalcitrance across all five processes (removals ≤ 29.70%) underscores a fundamental limitation of ∙OH and SO_4_∙^−^ based chemistries for this structural class. The only exception concerns the marginal improvement of UV/Cl for 2-MIB over GSM (28.02% vs. 26.49%). This improvement suggests that the larger, more polarizable Cl∙ may partially circumvent the steric barrier through enhanced selectivity for the tertiary C–H center adjacent to the hydroxyl group, a nuance that is masked in the other AOPs [40,46].

IPO and IPMP exhibit intermediate reactivity. IPO contains a carbonyl group but lacks extended conjugation, limiting both photochemical activation and radical addition, whereas IPMP benefits from its pyrazine ring with nitrogen heteroatoms, which provides some sites for electrophilic substitution. This reactivity hierarchy aligns with reported ∙OH and SO_4_∙^−^ rate constants for structurally analogous compounds [33,38,39] and offers a basis for estimating the UV-AOP treatability of emerging odorants based on their molecular features, with the order being conjugated systems > heteroaromatic rings > carbonyl-containing aliphatics > saturated cyclic alcohols.

### 3.3. Process Selection and Practical Implications

The tested UV-based systems exhibited distinct compound-dependent degradation patterns under the predefined operating conditions. UV/H_2_O_2_ achieved relatively higher removals of IPO and IPMP and the highest removal of GSM, although GSM degradation remained below 30%. UV/PMS performed comparatively better for CTA and 2,4,6-TCA, whereas UV/Cl produced the highest numerical removal of 2-MIB. However, these differences should be interpreted as treatment responses under the applied, non-optimized conditions rather than as evidence of intrinsic process superiority, because the oxidant and catalyst dosages were not normalized on an equivalent basis. The observed performance therefore reflects the combined effects of oxidant dosage, photochemical activation, reactive-species generation, and compound-specific reactivity.

A consistent relationship between molecular structure and degradation behavior was nevertheless evident. Odorants containing conjugated or aromatic moieties, particularly CTA and 2,4,6-TCA, were relatively susceptible to UV-based oxidation, whereas the saturated bicyclic alcohols 2-MIB and GSM remained recalcitrant in all systems. The favorable performance of UV/PMS toward CTA and 2,4,6-TCA may be associated with the electrophilic reactivity of sulfate radicals toward conjugated and aromatic structures [33,38,39]. In contrast, the higher removals of IPO and IPMP under UV/H_2_O_2_ may reflect the broad reactivity of hydroxyl radicals toward heteroaromatic rings and aliphatic side chains [36,38,39]. Although UV/Cl yielded the highest removal of 2-MIB, its limited absolute removal indicates that reactive chlorine species could not overcome the intrinsic resistance of the compact bicyclic structure.

Potential transformation products must also be considered [10]. UV/Cl may generate chlorinated or brominated products in waters containing halides [42], while UV/PMS benefits from longer-lived sulfate radicals (half-life ~30–40 μs vs. ~20 ns for ∙OH) [38]. Because transformation products and toxicity were not analyzed, parent-compound removal cannot be equated with mineralization or detoxification. Further evaluation of transformation-product identity, persistence, and toxicity is therefore required [18,34,40,41].

A rigorous engineering comparison would additionally require normalization of oxidant consumption, absorbed UV fluence, energy demand, chemical cost, and by-product formation. UV/TiO_2_ should be evaluated separately because TiO_2_ was applied as a heterogeneous catalyst rather than a stoichiometric oxidant. Its relatively low performance may have resulted from light attenuation and mass-transfer limitations. The methanol quenching procedure may also introduce uncertainty because methanol can react with ∙OH and SO_4_∙^−^ to form secondary radicals [47]. However, methanol was added only after sampling, so its influence on the irradiation-stage kinetics was likely limited. Future work should employ alternative quenchers or complementary techniques such as EPR.

Finally, the experiments were conducted in ultrapure water. Natural organic matter, alkalinity, and inorganic ions in real waters may scavenge radicals, attenuate UV light, and alter reaction pathways. The results should therefore be viewed as a controlled comparative screening rather than direct predictions of full-scale performance. Future studies should use representative source waters, normalized operating conditions, and energy- or fluence-based metrics.

## 4. Conclusions

This study investigated the seasonal occurrence of representative odorants across 20 surface water sources and evaluated the removal performance of six odorants under five UV-based oxidation processes, including UV photolysis, UV/H_2_O_2_, UV/PMS, UV/Cl, and UV/TiO_2_. Total odorant concentrations ranged from 8 to 496 ng L^−1^ in the wet season and from 5 to 184 ng L^−1^ in the dry season. 2-MIB was the predominant odorant, with a detection frequency of 95%, while the main associated water-quality indicators shifted from TP and DO in the wet season to NH_4_^+^-N and COD_Mn_ in the dry season. CTA showed the greatest removal efficiencies across the five UV-based processes, ranging from 74.66% to 95.03%. Moderate removal was achieved for 2,4,6-TCA and IPMP, with efficiencies of 43.92–73.17%, whereas IPO, 2-MIB, and GSM exhibited relatively limited removal under most treatment conditions. Among the evaluated processes, UV/PMS showed superior performance for CTA and 2,4,6-TCA removal, while UV/H_2_O_2_ exhibited better removal performance for IPO and IPMP. Overall, these findings provide a quantitative basis for further evaluating seasonal odorant occurrence and the compound-specific performance of UV-AOPs under more realistic water-treatment conditions. E-supplementary data for this work can be found in the e-version of this paper online.

## Figures and Tables

**Table 1:** Odor threshold concentrations (OTCs) and molecular formulas of typical odorants.

Odorants	Molecular Formula	Olfactory Thresholds (ng L^−1^)	Reference
2-MIB	C_11_H_20_O	5~10	[27]
GSM	C_12_H_22_O	1~10	[27]
TCA	C_7_H_5_OCl_3_	0.02~0.08	[27]
IPMP	C_9_H_14_ON_2_	0.4	[27]
IBMP	C_9_H_14_ON_2_	9.9	[27]
IPO	C_9_H_14_O	0.3~19	[28]
CTA	C_10_H_16_O	0.5~19.3 × 10^3^	[29]
Ionone	C_13_H_20_O	7	[29]

**Table 2:** Removal efficiencies of six odorants by different UV-based processes after 100 min.

Odorants	UV Photolysis and UV-Based Advanced Oxidation Processes				
	UV	UV/H_2_O_2_	UV/PMS	UV/Cl	UV/TiO_2_
IPO	12.28%	34.73%	24.76%	20.60%	4.73%
2-MIB	10.66%	25.04%	21.05%	28.02%	4.54%
GSM	9.55%	29.70%	16.25%	26.49%	26.08%
IPMP	45.06%	73.17%	58.24%	48.31%	43.92%
CTA	91.34%	89.53%	95.02%	95.03%	74.66%
2,4,6-TCA	58.53%	61.88%	66.62%	61.31%	56.97%

## Data Availability

The raw data supporting the conclusions of this article will be made available by the authors upon request.

## Supplementary Materials

The following supporting information can be downloaded at: https://www.mdpi.com/article/10.3390/toxics14080698/s1. Figure S1: Distribution of the 20 surface water sampling sites across Shandong Province, China; Figure S2. Schematic diagram of the UV photochemical reactor used in the oxidation experiments; Table S1: Automated solid-phase extraction (SPE) program for odorant preconcentration; Table S2: Mass-to-charge ratios (m z^−1^) and retention times of the target odorants in GC–MS analysis; Table S3: Pseudo-first-order rate constants (k, min^−1^) for the degradation of six odorants by UV photolysis and four UV-based advanced oxidation processes.
