# Supplementary material for: Seasonal Occurrence and UV-Based Oxidative Removal of Odorants in Surface Water: Insights into Structure-Dependent Reactivity
Source: Toxics. 2026 Aug 6;14(8):698. doi: 10.3390/toxics14080698 (PMC13517814; doi:10.3390/toxics14080698)
Supplement: Supplementary file 1 [file toxics-14-00698-s001.zip › toxics-4449199-supplementary.pdf]

Supplementary Material

Seasonal Occurrence and UV-Based Oxidative Removal of Odorants in Surface Water: Insights into Structure-Dependent Reactivity

Junkai Feng [a, 1], Congcong Li [a, 1], Hao Jiao [b], Xiaodong Xin [c], Ruibao Jia [a, *], Weiqiang Zhu [a, *]

a. School of Water Conservancy and Environment, University of Jinan, Jinan 250022, China

b. Jinan Municipal Engineering Design & Research Institute (Group) Co., Ltd

c. Shandong Provincial Water Supply and Drainage Monitoring Centre, Jinan 250101, China

[1] Equally contributed as co-first authors.

* Corresponding authors. E-mail address: stu_jiarb@ujn.edu.cn (R. Jia); stu_zhuwq@ujn.edu.cn (W. Zhu).

The following is included as supporting information for this paper:

Number of pages: 6

Number of figures: 2

Number of tables: 3

Figure S1. Distribution of the 20 surface water sampling sites across Shandong Province, China.

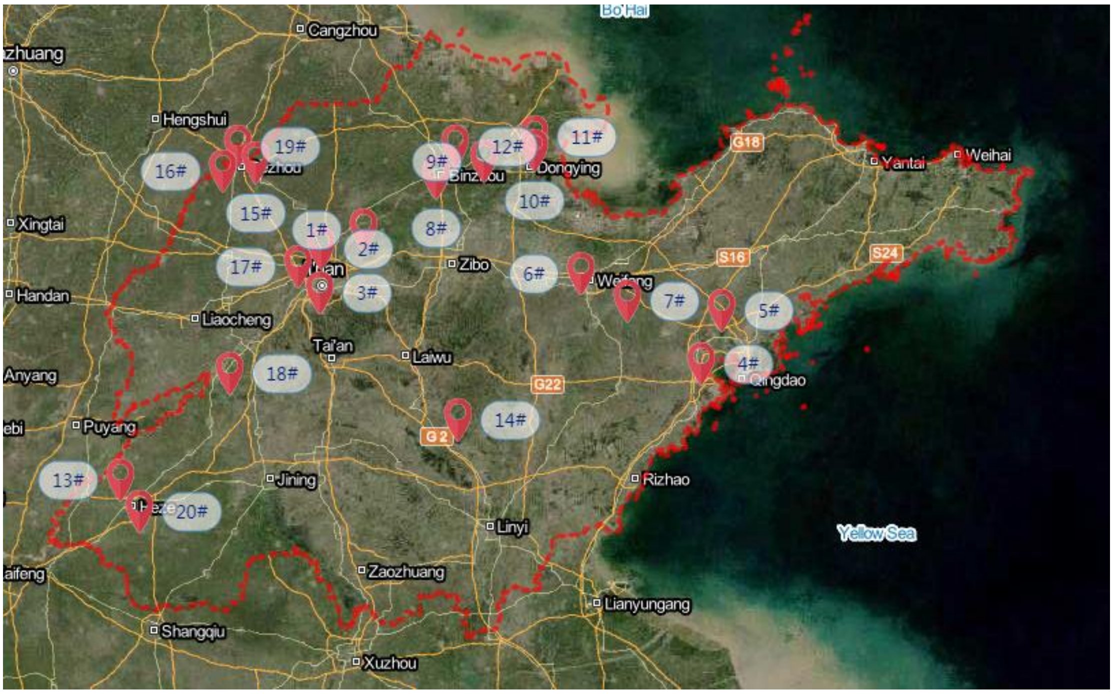

Figure S2. Schematic diagram of the UV photochemical reactor used in the oxidation experiments.

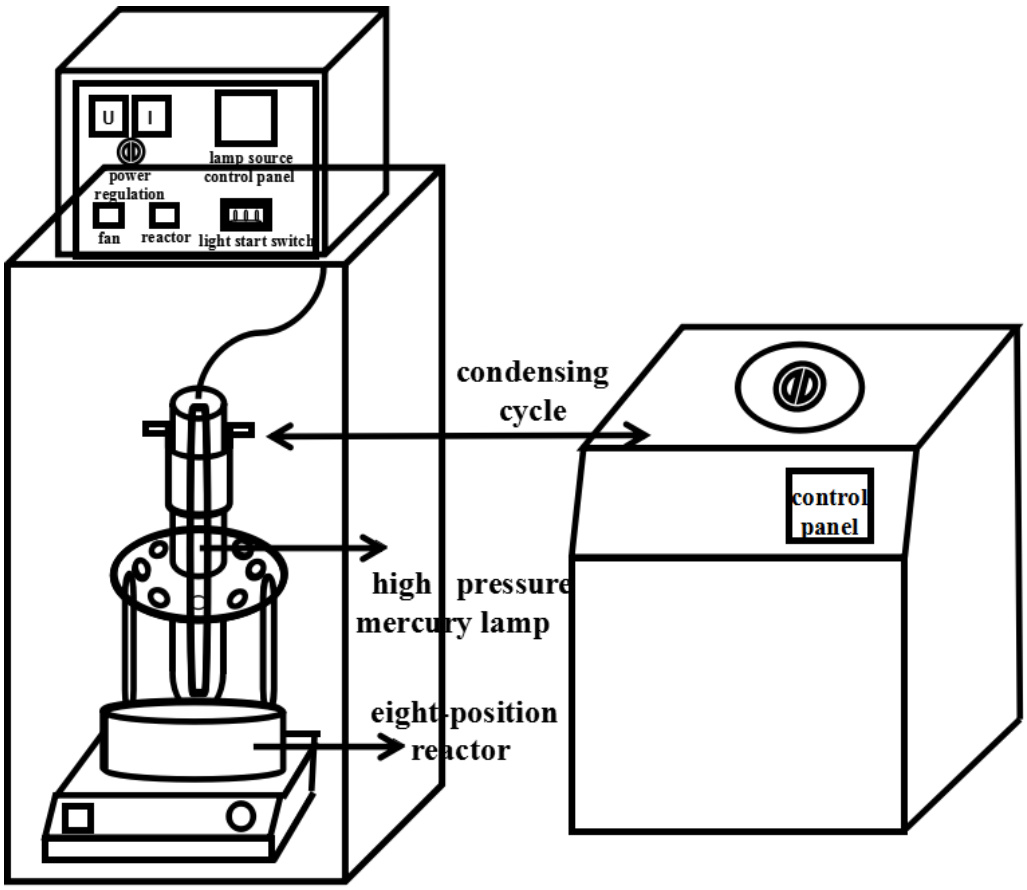

Table S1. Automated solid-phase extraction (SPE) program for odorant preconcentration.

Number	Order	Solvent	Discharge	Velocity (mL min^{-1} $°C^{-1}$)	Volume (mL)	Time (min)
1	Active	CH_3OH	Liquid waste 2	10	5	0.8
2	Active	H_2O	Liquid waste1	10	10	1.4
3	Sample	-	Liquid waste1	10	100	15.8
4	leaching	H_2O	Liquid waste1	10	5	0.8
5	Gas Push	-	Liquid waste1	10	10	1.6
6	Dry	-	-	-	-	25
7	Cleaning	CH_3OH	-	5	5	1.3
8	Cleaning	CH_2Cl_2	-	5	5	2.1
9	Eluting	CH_2Cl_2	Collect	3	2.5	1.4
10	Gas Push	-	Collect	3	10	3.9
11	End	-	-	-	-	-

Table S2. **Mass-to-charge ratios (m z^{-1}) and retention times of the target odorants in GC–MS analysis.**

Olfactory substances	Mass to charge ratio (m z^{-1})	Retention time (min)
2-MIB	95	10.67
GSM	112	14.53
IBMP	124	10.52
IPMP	137	8.94
IPO	82	9.54
CTA	152	11.32
2,4,6-TCA	195	13.23
2,3,4-TCA	210	16.21
2,3,6-TCA	210	13.93
Ionone	177	15.74

Table S3. **Pseudo-first-order rate constants (k, min^{-1}) for the degradation of six odorants by UV photolysis and four UV-based advanced oxidation processes.**

UV -AOPs	Olfactory substances					
	2-MIB	GSM	IPO	IPMP	CTA	2,4,6-TCA
UV	1.44×10^{-3}	9.06×10^{-4}	1.09×10^{-3}	6.59×10^{-3}	2.31×10^{-2}	9.25×10^{-3}
UV/PMS	2.40×10^{-3}	1.87×10^{-3}	1.42×10^{-3}	8.77×10^{-3}	3.07×10^{-2}	1.10×10^{-2}
UV/H_2O_2	4.23×10^{-3}	2.33×10^{-3}	2.98×10^{-3}	1.30×10^{-2}	2.15×10^{-2}	9.42×10^{-3}
UV/Cl	2.54×10^{-3}	3.00×10^{-3}	2.75×10^{-3}	6.94×10^{-3}	2.67×10^{-2}	8.82×10^{-3}
UV/TiO_2	5.14×10^{-4}	4.00×10^{-4}	2.59×10^{-3}	6.59×10^{-3}	1.23×10^{-2}	7.98×10^{-3}
